# On the Physiology and Psychology of Apparitions, Dreaming, &c.

**Published:** 1848-10-01

**Authors:** 


ART. III.—*The Night Side of Nature.* By CATH. CROWE. 2 vols. Newby. 1848.

*Des Sciences Occultes; ou, Essai sur la Magie, &c.* Par EUSEBE SALVERTE. A Paris. Chez Bailliére.

*The Philosophy of Magic.* From the French of Eusébe Salverte. By A. T. THOMSON, M.D. 2 vols. Bentley. 1846.

*A World of Wonders.* Edited by ALBANY POYNTZ. Bentley. 1845.

*Communications with the Unseen World.*

*On Dreams, in their Mental and Moral Aspects.* By JOHN SHEPPARD. Jackson and Walford. 1847.

*The Philosophy of Mystery.* By WALTER COOPER DENDY, late President of the Medical Society of London, &c. London. Longman and Co.

THE question regarding those psychological phenomena which (in reference to their prevalent association with the visual sense,) have been termed ghost, spectre, phantom, eidolon, shadow, shade, has been, especially in the mediæval period, a subject of much obscurity and speculation.

Even in later and more rational times the spiritualist, in his anxiety to avoid the Scylla of materialism, has been engulphed in the whirlpool of abstract metaphysics; while the sceptic, blushing to acknowledge aught without the comprehension of his sovereign reason, has blinked even the miracles of Holy Writ, in his aim to find a material or palpable cause for all.

Wierus, Bodinus, Raymond, Lully, Beaumont, Berthogge, Baxter, Aubrey, Glanville, and Moreton, have long since doled out to us, with marvellous credulity, their mystic legends. In later times, Brown, Reid, Hibbert, Ferriar, and Dugald Stewart, have written very learnedly on the philosophy of mind and its eccentric derangements: and later still, Salverte and Brewster have shed the gleams of science on these paths, and unfolded to us the whole realm of natural magic. These may be termed the masters of the visionary, the mental, and the mechanical schools.

From the dark ages, we can hence trace the doctrine of the occult sciences progressively declining as the human mind emerged from the trammels of superstition and priestcraft, and the light of pure philosophy beaming forth to illustrate the wonders of the creation.

We have now no professed oneirocritic, no Artemidorus to interpret our dreams, no Lilly to cast our nativities, no Hopkins to ferret out a witch. We had believed, therefore, that the world of shadows had gradually faded away, when, lo! even in this matter of fact era, a female spiritualist of a novel genus, appears in the arena of psychology—one who, adopting and exaggerating the notions of Lucretius and Paracelsus, and leaving even Essenmayer and Reichenbach in the lurch, essays to prove not only the visible but the palpable existence of a real ghost. The metaphysical spiritualism of Berkeley is of course annihilated, the material psychology of Paley, and the mental corporeality of Priestley are fairly distanced. Idealism itself is a mere idea—everything, even spirituality, is real. There is no such thing as *subjective* phantasy—the ghost, the vision, the dream, are all *objective*. Yet is not Mrs. Crowe yet satisfied. She is evidently leaning even to the *projective* theory of ghosts: whether they be termed the astral spirit of Paracelsus, or the eidolon or chrysalis of Lucretius, or the —— something else of Reichenbach: it is all real stuff. It seems, however, that the phantom is not of so high a specific density as flesh and blood; it must be, as Baxter terms it, "matter of a less spissitude."

This is materialism with a vengeance—endowing the soul with new faculties. It may have a special endowment of deuteroscopia, an acute faculty of discerning the "phosphorescent shadow of a body living or dead: or it may *project* itself into the sphere of another's vision; or it may even possess the power of speech."

Now, if this be not the Elizabethan, it may truly be termed the Victorian age of woman. One has essayed to teach the lieges the principles of political economy: another, of yet deeper blue, has

> "Into the heaven of heavens—presumed
> An earthly guest, and drawn empyreal air."

And now a third appears, of very sable hue, to analyse the mighty mind of man: and not only to draw aside the veil with which creative wisdom has enfolded the mysteries of the immortal spirit, but to people the earth —ay, even without the stones of Deucalion and Pyrrha, with a great and wondrous legion of shadows. Even though not often seen, before our eyes, in our very presence, stands a corps of horrible shades, that only await a fitting opportunity—that is, a condition of our own souls congenial to theirs—to render themselves visible, audible, and tangible to our senses.

Happily, however, these states are very rare, and these half-angel visits are few and far between.

Now we implicitly believe, that these phantasies exist but in the mind's eye of the writer, yet as their record may so work on weak and sensitive minds, which experience a morbid delight in brooding over bloody heads and naked bones, as to induce many baneful effects, we feel it our duty to attempt to point out their fallacy.

We have, therefore, placed together two works, the very antipodes of each other, yet bearing one feature of close resemblance, the reality of their ghosts. According to both the mind is, to a degree, passive—the lady believing that it is acted on by the objective soul of the phantom—the Frenchman terming all visions spectral illusion, induced by the mechanical imposture of priests and magi. So much, indeed, is Monsieur Salverte in love with matter-of-fact, that the accepted miracles of Holy Writ are problems as easily solved as the most simple trick of a modern conjuror.

There is, it is true, not much of psychology in this: but the author has set himself, and that successfully, to found a sort of chapel of ease to the science, by his laborious researches into the realms of natural magic from the earliest period, and thus has negatively at least illustrated psychology by explaining very lucidly and scientifically the causes of these phenomena which the philosophy of mind had failed to do. He has, therefore, imparted a deeper interest to the legends of antiquity, many of which we have been wont, in default of his book, to term fabulous and mythological. Thus the work forms a valuable adjunct to Sir David Brewster's natural magic.

The author has thrown a political and religious interest over his book. He even refers despotic and enslaving government to magic and imposture: alluding to the *fixed form* of civilization still prevalent in heathen Asia, and the *perfectible* form in Christian Europe, that has enlightened the once dark minds of the human race: thus, as his translator says, in reference to the pious frauds of the dark ages, " throwing open the gates of the ancient sanctuaries."

We read thus:—

> " From the most ancient time, men of superior intellect, desirous of enthralling the human mind, have adduced miracles and prodigies as the certain proof of their missions, and as the inimitable works of the deities whom they revered. Seized with terror, the multitude have bent beneath the yoke of superstition: and the proudest man has touched the steps of the altar with his humbled brow."

While we add our unfeigned eulogy of the deep research displayed, we cannot but smile at some little scraps of credulity even in our *rational* author. For instance, he believed we can charm serpents by a mere touch.

With regard to our criticism of the lady's visionary pages, we confess our gallantry has somewhat wavered. But the Amazon, when she buckles on her breastplate, does in reality unsex herself: she must not wonder, when she throws down the gauntlet, that her challenge is accepted. We have, therefore, a crow to pick with Mrs. Crowe.

If the authoress had presented her amusing and really curious anecdotes merely as a Radcliffian romance, or book of wonders, our criticism had, of course, been silent. But when she presumes to philosophize (we suppose she would term it), even though she apes humility and the form of proposition, affirming not only the substantial reality, but the chemical qualities of ghosts of every sort—not the mechanical spectre, be it told, but the chemical embodiment of the spirit or soul, we deem it but justice to society to criticise her notions.

And how does she word her challenge?—Thus: "Of the explanation of psychological phenomena, we are yet in a state of entire and wilful ignorance." Then the works of Locke, Brown, Reid, and Stewart, go for nothing. "Science in this country has put it aside as beneath her notice!" Then Salverte and Brewster have in vain endeavoured to draw aside the veil of imposture and expose the frauds of thaumaturgy. These sentences show plainly how narrowly or blindly Mrs. Crowe has studied her subject ere she issued her libel on science. Like Democritus, one would think she had put out her eyes, that she might not see; and although she professes to stimulate inquiry rather than to teach, she should, at least, have perused in real good faith, if not acknowledged, those established works, which have been written on the mystical union of body and spirit with an equal regard to metaphysical physiology and the sacredness of revealed truth. She would not then have been more bigotedly determined to consider science but a blind guide, and affirmed all mysterious incidents *supernatural*, for such, in spite of her denial, her explanations must be deemed, than Hibbert, Ferriar, Brewster, and Salverte have been, to term all illusion. She would have blushed to affirm that psychology cannot be elucidated "by the most acute intellect, by the most powerful logic;" or that it is "equally irreducible within the present bounds of science."

Now, we have ever regarded psychological physiology as a subject of deep solemnity, and after much analogical and analytical study, are prepared to stamp Mrs. Crowe's rhapsodies as a very superficial and erroneous compilation of arguments, if such they may be termed. In her lucubrations on the soul's nature, she has wofully slighted the importance of the brain, the great organ by which alone its workings can be made known to the intellect. She has blinked the mystical union, and yet, while she embodies the spirit in a shadow of light, she has sadly misunderstood the mechanism of mind, the only earthly medium of our intercourse with the world of spirits.

Brain, however, (we quote from the Philosophy of Mystery,) can no more be considered as mind itself than *retina* sight; or than the sealing wax can be identical with the electricity residing in it. Brain is the habitat of mind, the workings of which cannot be indicated without it; for as the material world would be intact without a sense, so there can be no *mental evidence* of mind without a brain, which is indeed the sense of the spirit. Thus, without adopting the creed of the *Hyloist*, the moderate materialist, that the mind cannot have, during the life of the body, even a momentary existence independent of matter, I believe that when this matter is in a state of repose, mind is perfectly passive *to our cognizance.*

Ida, a devotee, then asks the philosopher Evelyn, (page 183), "Does not this imply the office of a gland, that brain is the *origin* of soul, and that its function was the *secretion* of *thought.*" He replies—" Such is the timid error of the mere metaphysician; for if there *be* secretion, it is the soul that directs."

Agreeing with this exposition, we will ourselves take, further, the analogy of a sense. As the globe of the eye must *be* before there is sight, the brain must *be* before there is intellect. Light gives sight to the eye: soul intellect to the brain.

Again, the study of philosophy is nature and nature's known laws. If we lean for a moment to credence in a modern miracle, philosophy may close her book: unless, indeed, we consider miracles in the same light as Salverte.

> "Miracles and marvellous events, equally in connexion with supernatural influence, are often wonders worked by men. Whether they pretend that a benevolent or a terrible divinity employs them as instruments: or whether by the study of the transcendental sciences they assume that they have subjected to their empire spirits endowed with some power over the phenomena of the visible world."—Vol. i., p. 9.

But on revealed truth and the immaterial nature of the mystical essence within us, we may not lightly discourse: they are of too holy a nature to be submitted to the test of philosophical speculations, and especially to the fancy of enthusiastic rhapsodists. When, therefore, the physiologist has explained, according to acknowledged laws, those phenomena which, without them, would be inexplicable, he must rest satisfied; the emancipation of the soul is " a sublime truth, resting on proof far more sacred than may ever be elicited by the working of man's sovereign reason."

Philosophy stops when she *cannot* demonstrate or explain. But our authoress is a deep one—she explains everything, ending her paragraph thus: " this admits no other explanation;" " as who should say, I am Sir Oracle." Yet she terms the rational pathologist presumptuous, when he alludes to the result of a peculiar condition of the mind of the being in the flesh.

It is conceded that disease, intoxication, &c., *may* induce morbid or debilitated states of the brain; but this, it seems, is merely rendering it fit to be influenced by spirits, good and bad, who impart to it warnings, free knowledge, &c.; and yet Mrs. Crowe is willing to wait on, before she quite believes the disentanglement of soul.

We have a *hint,* that the brain being thus diseased or asleep, the soul flits away, (the hypothesis of good Sir Thomas Brown, long since asleep himself,) and tells the brain of its wanderings on its return: but we must still wait for the full development of animal magnetism, then

> " We shall be shown by Mesmeric light that we are indeed children of God !"

And yet all this *may be* the effect of disease, which, therefore, must render the mind itself more perfect, at least more comprehensive.

Regarding these rhapsodies of Sir Thomas Brown—the flight of soul —we may here observe, that Mr. Sheppard is to a degree a proselyte:

"We are somewhat more than ourselves in our sleep, and the slumber of the body seems to be but the waking of our souls."

To illustrate the activity of the spirit in the dream, he adduces the *decies repetitiæ* stories of the Kubla Khan of Coleridge, the Creation of Cœdmon the Anglo-Saxon, the compositions of Condorcet, Franklin, Cardan, and Tartini, and also the notions of Addison, of Bishop Newton, and Abercrombie; to these he might have added, Condillac, La Fontaine, Voltaire, Haycock, and McKenzie.

We do not doubt these facts, but we differ as to definition. The state of mind was reverie, not sleep; "intense thought," as Rosencrantz writes, "without images." In the words of Hoffbauer, "the intensity predominates over the extensity."

"Under this condition, the influence of external objects is often for a time lost. The retina may be struck by a ray, or the membrana tympani by a vibration, but the mind shall fail in its perception, no internal *impression* being made."—*Philosophy of Mystery*.

This state of the cerebral faculties Mr. Sheppard identifies with dreaming, and adduces it to controvert the views of materialism. And yet he does not altogether dissent from Hartley, and Paley, and Johnson—nay, he even seems to agree with the Pythagorean and Platonic philosophy, "that the human soul has an interior luciform æthereal body, which remains united to it after death." Paley and the rest were, therefore, *devout materialists.*

"The archdeacon's opinion that we should have a substantial resurrection, is founded on New Testament evidence, and is expressed in his discourse on a future state. The apostle's simile of the wheat implies a *death* of the grain: it dies, but there is *no remodelling*, for it is the germ that lives and grows: so, although the body may not be restored, there is a development of *its* germ in the transit or resurrection of its spirit. In the words of Ecclesiastes, 'Then shall the dust return to the earth as it was, and the spirit shall return unto God who gave it.'"—*Philosophy of Mystery*, p. 187.

Now, if *we* might interpret, it seems that the new wheat is the *child*, not the dead body. If conception occur, the fœtus is developed and grows. So, if the seed be sown, the vegetable ovum, or fœtus, is also developed and grows. The changes of the butterfly might be cited as close to St. Paul's analogy: the things are very similar; the germ of the fly, and of the wheat, existing in the ear of corn and in the chrysalis.

To return to the Night-Side from this transient elucidation.

If we can dive deeply enough into Mrs. Crowe's spirit, it would seem to be essential, ere it perceives that it should assume a palpable form. How, then, can we explain *foresight*, the anticipation of that not yet existing? Oh, with the greatest possible ease! "We see not only what never was, but what never will be." This beats the critic hollow: and to illustrate (?) this, Mrs. Crowe adduces as an analogy her own visions after eating bad bread! Really this is such a paradoxical jumble that we smile in pity while we read it.

This is, of course, *subjective* phantasy.

Again, in referring to the hackneyed case of the bookseller of Berlin, Mrs. Crowe is very brusque. She tells us everybody knew this to be morbid. No doubt; and so they do a host of other cases, which, if she deigned to notice, she *must* confess were not objective, or projective of a spirit from its body: nor would she argue that a *morbid* state induced that exalted power of sense which imparted a deeper insight into nature's wonders than a healthy condition of an organ. Yet the case of Nicolai was as seemingly supernatural, and as rationally explicable, as possible—and in the authoress's own words: "A thousand negatives cannot overthrow one palpable fact."

But Mrs. Crowe, as we have before observed, is not contented with this confession, even with her own *objective* hypothesis—the *real* presence of the shadow or the ghost—she must call in Dr. Ennemoser, with his theory of the *projective*—a sort of universal law of polarity or intercommunication of beings—the *rapport* of the Mesmerist. We are, however, somewhat pacified to find this author make up for his polar dogmatism by confessing, that in their earthly combination, the soul and body cannot act independently of each other: the spirit can only perceive through the body.

Of the myriads of dreaming prophecies unfulfilled, Mrs. Crowe is very shy; but if her dreams are projections of spirits, why come they in vain? We are told that the grandmother of Councillor Schwartz, of Heidelburg, communicated to him in a dream a Greek manuscript, regarding his destiny, which, although then almost ignorant of the language, he read fluently;—why not in German? Mrs. Crowe confesses this is very odd. Then the son of Dante was told in a dream, by his father's spirit, to look for a missing Canto behind a panel, where it was found! What shallow premises; who can affirm that he was not aware of this. These are fair samples of the lady's credulity.

Now, few have ever doubted that ghosts and spirits *may exist*—that their appearance, as well as the whole mass of mysterious legends scattered through the pages of literature may be so far explained by the workings of our living and embodied minds. The manifestations of mind through brain and matter are so demonstrable, that we do not hesitate to throw back the *materiel* of phantoms on the multiformity of our own ideas and thoughts. At least, a metaphysical or psychical ghost (and is there any other?) is *an intense idea.*

"'Do you think it strange,' observes Evelyn, in the 'Philosophy of Mystery,' 'that a ghost should appear, fleshless and shadowy, without some supernatural influence? Be assured that the only influence exists in the sublime and intricate workings of that mind, which in its pure state was an emanation from the Deity: which is only shadowed by illusion while in its earthly union with the brain; and which, on the dissolution of that brain, will again live uncombined, a changeless and eternal spirit.

"'It is as easy to believe the power of mind in conjuring up a spectre, as in entertaining a simple thought: it is not strange that this thought may appear embodied, especially if the external senses be shut: if we think of a distant friend do we not *see* a form in our mind's eye, and if this *idea* be *intensely* defined, does it not become a phantom?'"

Again—

"Although the ghost will not appear to tell us what *will* happen, it may rise, and with awful solemnity too, to tell us what *has* happened. Such is the phantom of remorse, the shadow of conscience—which is, indeed, a natural penalty—a crime that carries with it its own consecutive punishment. Were the lattice of Momus fixed in the bosom, that window through which the springs of passion could be seen, there would be, I fear, a dark spot on almost every heart—to quote the Italian proverb—'A skeleton in every house.' Brutus, and Richard Plantagenet, and Clarence, and Macbeth, and Manfred, and Lorenzo, and Wallace, and Marmion are but the archetypes of a very numerous family in real life, for Shakspere, and Byron, and Schiller, and Scott have painted in high relief their portraits *from* the life."

Mrs. Crowe, however, spurns scientific or physiological explanation, while she leans blindly to everything which *she* is pleased to call a fact, admitting, at the same time, that the facts she adduces "have really no scientific value." She is at present determined that they *shall be* inexplicable; and directly prognosticates that the time will come when "they will be reduced strictly within the bounds of science."

Now, *we* believe that in all these cases there is a very high value in a medical point of view—they are a precious piece of symptomatology, indicating, in very potent language, the pathology of brain and mind. We will again briefly adduce the argument of Evelyn.

In allusion to second sight, he says—

"The efforts of the seers, or the mysterious ordeal to which they submit themselves, are often so painful that they gaze with strained eyeballs, and fainting occurs as the vision appears. When the dark hour is over they will exclaim, with Mac Aulay, 'Thank God the mist hath passed from my spirit.' Indeed, Sir Walter observed in those who presumed to this faculty, 'Shades of mental aberration which caused him to feel alarmed for those who assumed the sight.'"

Again—

"My friend, Dr. James Johnson, has told me of a gentleman of great science, who conceived that he was honoured by the frequent visits of spectres. They were at first refined and elegant both in manners and conversation, which on one occasion assumed a witty turn, and quips, and puns, and satire were the order of the evening: so that he was charmed with his ghostly visitors and sought no relief. On a sudden, however, they changed into demoniac fiends, uttering expressions of the most degraded and unholy nature. He became alarmed, and depletion soon cured him of his phantasy."

We might cite very many analogous instances of the influence of black blood about the brain and heart, from Conolly, Abercrombie, &c., as well as the cases of Count D'Olivarez, Earl Grey, Lord Castlereagh, the Scotch Lawyer of Scot, the Martyr Philosopher in the "Diary of a Physician," but we have already written enough. We will merely refer to the cases in the "Philosophy of Mystery," of Ariosto, Rousseau, Cooper, Collins, &c., to prove the frenzy so often allied to genius. "The laurel," as Ida observes, "contains more poison than that of prussic acid in its leaf."

The *rationale* of dreaming may be here appropriately illustrated.

Mr. Sheppard's book on this especial subject contains a train of very candid metaphysical reasoning, *apart from physiology*. It therefore leans too much to abstract deductions. His allusions to the utility of dreams in checking atheism and conversion of erring souls, are the work of a well regulated and sensible mind, but in a psychological journal it is essential that pathology should not be thus hoodwinked, we therefore proceed to quote from the "Philosophy of Mystery" Evelyn's pathology of dreaming:—

"1. A susceptibility of influence.
"2. The influence itself.
"3. The effect of this influence.

"And these I call the *predisposing*, the *exciting*, and the *proximate* causes.

"1. The brain is brought to this susceptibility by excited temperament, study, intense and undivided thought; in short, by *any* intense impression.

"2. The influence or excitement is applied; congestion of blood producing impression on extremities, or origin of a nerve at the period of departing or returning consciousness. At these periods, the blood changes, and I believe, *as it changes*, the phenomena of mind, as in the waking state, obey these changes; rational or light dreams being the effect of scarlet blood; dull and reasonless visions and night-mare, that of crimson or black blood.

"3. The effect of this influence is recurrence of ideas. *Memory:* more or less erroneously associated, as the blood approximates to the black or scarlet state, or as the brain itself is constituted.

"Now, it is essential to the perfect function of the brain, not only that it shall have a due supply of blood, but that this blood shall be of that quality we term *oxygenated.* If there be a simple *deficiency* of this scarlet blood, a state of sound undisturbed sleep will ensue, slightly analogous to the condition of syncope or fainting. This may be the consequence of any *indirect* impression, or the natural indication of that *direct* debility which we witness in early infancy, and in the 'second childishness and mere oblivion' of old age. But this deficiency of arterial blood may be depending on a more positive cause, *venous congestion*, impeding its flow; for in sleep the breathing being slower, the blood becomes essentially darker. Even arterial blood itself will become to a certain degree *carbonized*, by *lentor* or stagnation. Venous congestion and diminution of arterial action are not incompatible; indeed, Dr. Abercrombie reasons very ably on their relative nature, implying the necessity of some *remora* of venous circulation to supply that want or vacuum which the brain would otherwise experience from the deficiency of the current in the arterial system. Thus will the languid arterial circulation in the brain, which causes sleep in the first instance, produce, secondarily, that congestion of blood in the veins and sinuses which shall reduce it to disturbed slumber, and excite the disease. May we not account, on this principle, for the difficulty which many persons experience in falling into a *second* slumber, when they have been disturbed in the *first.*'

We were startled *in limine* at Mrs. Crowe's psychology. Let us now contemplate the destinies of her ghosts. In the profane myths of the ancients, we are introduced to *three* ghosts of a body, itself being "quietly inurned." The spirit went up to heaven—the soul to Hades—the shade wandered about the tomb or over the earth.

Our own modern creed has hitherto been satisfied with *one* immortal spirit—the soul (let the Greeks call it what they will, θυμος, νους, φρην, or ψυχη) the spiritual body of St. Paul—the fundamental life (!) of the reverend visionary Townsend. But Mrs. Crowe, the unconscionable, must have two, the spirit and the soul—the latter being subject to the former, in which, according to another dreamer, dwells the conscience.

It is evident, however, that Mrs. Crowe misinterprets. *Pneuma*, which she calls soul, is the *breath of life* inspired or breathed into man at his creation—a vital, not a psychical property. Yet, in the Corvine psychology, this breath is a conscious agent—and like Ariel, the "naughty spirit," is often running wild, and then, in consequence of this disobedience, becomes degraded and debased. And these have a palpable and real existence; and yet Mrs. Crowe wonders that they are so seldom seen. She has, however, a reason for this. The reader will probably conjecture that is the cause advanced by the sage Mr. Puff, the Critic—"because they are not yet in sight." No such thing. It is because either we or the ghost are *deficient in our projectile energy*, and therefore they are invisible. Seldom seen!—why ghosts are seen by some visionaries or dreamers every day and night: some never lie down but they dream, or in other words, see phantoms.

Now, we think these matters of ghosts may be illustrated and explained without the science of projectiles.

For the reception of especial impressions, the mind must, of course, be predisposed. We will, therefore, refer to these *moods*, which Evelyn, in the Philosophy of Mystery, adduces as the predisposing causes of illusion:

" *Temperament*—Credulity, enthusiasm, superstition, timidity, imagination, poetic frenzy.

*Excitement*—Sympathy, exalted joy, deep grief, love, hatred, protracted anxiety, delirium of fever, delirium of alcohol, delirium of narcotics, exhaustion, disease of the brain.

The mind, then, endowed with any of these temperaments or excitements, is in a state of acute or morbid sensibility, and more or less intensely prone to the calling up of ghosts, which may be arranged in two distinct classes :—

GHOSTS OF THE MIND'S EYE, OR PHANTASMA.

| | |
|---|---|
| Illusive *per*ception, or ocular spectra . . | *Conversion* of natural objects into phantoms. |
| Illusive *con*ception, or spectral illusion . . | Creation of phantoms. |

GHOSTS OF THE EYE, OR OPTICAL ILLUSION.

| | |
|---|---|
| Atmospheric . . . | Refraction.<br>Reflection. |

Gases.
Lenses and mirrors.
Disease of the eye.

In the first class there is no real or palpable object, or if there be, it is not what it appears : the illusion is but the reality of romance, depending altogether on excited or disordered conditions of the mind; the source, therefore, of bright or gloomy phantoms, as the mood may be.

The second class, which are spectres or ghosts of the eye, may be scientifically explained by the laws which govern the *material world*. The objects themselves exist, and are exactly as they appear. The philosopher regards them as interesting exceptions to general rules, from *peculiar* combinations of *natural* causes. The unlearned will term them preternatural *phenomena*, simply because they are of uncommon occurrence. But which among the works of creation is not a phenomenon ? We may think we know a law of nature, but can we analyse it. Novelty and magnitude astonish, but that which is familiar excites not our surprise. We gaze with delight on the progress of an eclipse; we watch with wonder the eccentric course of the comet; but we look on the sun in its meridian glory with a cold and apathetic indifference. Yet do they all alike display Divine omnipotence, and the expansion of a vegetable germ, the bursting of a flower, is as great a miracle as the overwhelming of a deluge, the annihilation of a world. To discriminate between these two classes is not difficult : we may prove their nature by simple experiment. Optical illusions will be *doubled* by a straining or altering of the axes of the eyes; and by turning round, as they are removed from the axis of vision, they will disappear.

So, indeed, will those of the second class, which are *real* objects converted into phantoms by mental excitement or disorder. But in the purely *metaphysical* ghost or phantom, the change of position or locality will not essentially dispel the illusion (the spectrum following, as it were, the motion of the eye), because it exists in the mind itself, either as a faint or transient idea, or as a mere outline, fading, perhaps, in a brighter light, or as the more permanent and confirmed impression of insanity, unchanged even by "brilliant glare," or from the day-dream of the castle-builder to the deep and dreadful delusion of the maniac.

Of the atmospheric phantoms, or *lusus naturæ*, what myriads are presented to us—such as the snow-storm of Languedoc, the arctic phantasmagoria, the parhelia or mock suns, the aurora, the fata morgagna, the Calenture, the spectre of the Brocken, and a host of others scattered through the pages, especially of Dr. Thomson's volumes of Salverte—a work rich also to profusion in illustrations of the illusions of lenses and mirrors, as well as very acute and logical definitions of miracles, prodigies, allegory, fable, figurative expression, poetic fiction and metaphor, in which so many mysterious records have their source; and moreover, of ventriloquism and phonic mechanism—so that, as we peruse Salverte, we have, as Mrs. Crowe somewhere writes, an *embarras de richesses!*

Then, as to the chemical explanation of the gaseous ghost, Mrs. Crowe is, of course, a proselyte to Palingenesy, believing that—

"——— chemists, from a rose's ashes,
Can raise the rose itself in glasses."

She would pin her faith to the creed and stories of Astrophel, in the "Philosophy of Mystery," some of whose words we quote:—

"The Parisian alchemysts of the seventeenth century demonstrated this mystery, and raised a phœnix from its ashes. They submitted to the process of distillation some earth from the cemetry of the Innocents, during which ceremony they were scared by the appearance of perfect human shapes, struggling in the glass vessels they were employing. Dr. Ferriar thus deposes:—'A ruffian was executed, his body dissected, and his skull pulverized by an anatomist. The student who slept in the chamber of the experiment, saw in the night-time a progressive getting together of the fragments, until the criminal became perfect, and glided out at the door!'

"The apparitions of souls departed do, by the virtue of their formative plastic power, frame unto themselves the vehicles in which they appear out of the moisture of their bodies. So ghosts do often appear in churchyards, and that but for a short time, to wit, before the moisture is wholly dried up.

"'Such are those thick and gloomy shadows damp,
Oft seen in charnel vaults and sepulchres,
Lingering and sitting by a new-made grave.'

And we read in the chronicles, that 'during the time that the antients *burned*, not *buried* their dead, there was no such appearance of ghosts as is now."

On these or similar dark mysteries, Mrs. Crowe cites stories from her favourite German. Yet here she often refers the warping of exhalations into the forms of ghosts to disease or superstition: and then flies off at a tangent from Ennemoser's polarity to the Socratic, Platonic, and Brunonian theories, of the loosening of a spirit, and its unfettered wanderings, until she becomes absolutely bewildered—a condition in which we are doomed to bear her company.

For a full exposition of illusions produced by lenses and mirrors, we must refer our readers to the treasury of Salverte, and glance at Mr. Dendy's remarks on the illusion of disordered vision:

"In many cases of disordered sensibility of the retina, it is influenced by the minute *villi*, or vessels, in the tunics of the eye. In the case of its exhausted energy usually accompanied by night blindness, where there is no vision but in a strong light, the floating specks, termed *muscæ volitantes*, often become so numerous as to impart a notion of films floating in the aqueous humour, or before the cornea. It is a curious question, in what portion of the retina the spectra of *muscæ volitantes* are excited. They appear in or near the axis of vision; but as they do not interrupt the visual rays from *material objects*, it is possible they may arise on that spot considered to be destitute of vision, with regard to *external* impression. Or they may be produced by detached parts only of the objects, which impinge on the retina, reaching the brain. If the integrity of certain of its fibres, which, by converging, form the optic nerve, be destroyed, distorted or imperfect objects will be represented. This speck may be a musca volitans.

"The form of disordered vision, occurring so often in nervous persons, or resulting from close application to study, does not often appear to depend on a *turgid* condition of the vessels of the choroid or retina. It is usually relieved more by tonics than depletion, and very strange illusions of sight will sometimes be produced by depressing medicines, especially antimony. Yet these specks appear to be floating *before*, and often at some distance *withoutside* the eye. Therefore, we may believe that excited images may also appear before the retina, palpable."

Of cases of presentiments and warnings, Mrs. Crowe is very profuse, but they are calculated to inspire excessive fear in sensitive minds—indeed, to realize the catastrophe by the severe mental shock which is imparted. The stories of Miss Lee and Lord Littleton are illustrations of this influence, and we might refer for further proof to the works of Travers, and especially of Sir A. Crichton, on mental diseases.

Presentiment, it seems, is with Mrs. Crowe analogous to instinct. Of this, we have a very forcible illustration in a dog, which was very reluctant one day to take his usual walk: but he was driven out and was soon torn to pieces by a great savage dog just landed. This story is followed by an apology for the assimilation of animal instinct to human foresight, and an affirmation that instinct is far beyond our comprehension. But is it not clear that instinct is an impress of the Deity on irresponsible beings—one step higher than the habits and propensities, so to write, of plants. Man is left to act in his own faculty of judgment, while, to a certain degree, the brute is automatic, and impelled to act—*an effect of polarity*, Mrs. Crowe would affirm. The cited story of Sir Evan Nepean is, of course, an illustration of this polarity: he one night got up in a fit of restlessness, and walked through the Park to the Home Office, where he read that a reprieve had been sent to York for two coiners. But he found it had *not* been sent. By dint of much exertion, the reprieve arrived just in time to arrest the execution.

Mrs. Crowe has a redeeming point—she qualifies her horrors by displaying one very comfortable and polite creed, when writing on warnings and attendant spirits—viz., that we are free agents only when obeying dictates of virtue, and in bondage while following vicious propensities. But if this be, we are of course ourselves innocent, and the demon tempter should be punished, and not we, for the delinquency.

Some of the stories of dreaming are shallow beyond the general mark. A Dr. W., of Glasgow, dreams of crossing a moor on horseback, and being attacked by a bull, from which he escaped by getting to a spot inaccessible to the animal. In the morning, in obedience to a professional summons, he did cross a moor, was attacked by a bull, and escaped by getting to the spot described in his dream.

A butcher, at Holytown, dreamed that, on a certain spot, two men in blue clothes cut his throat. On the following day his duties led him by the spot, but he had the prudence to take a companion. He there saw two men in blue, who, on their approach, ran away.

Now, why did not these people stay at home, out of danger? "Oh," says Mrs. Crowe, "the spirit saw what was impending, but the intellect did not accept the warning!" A very lucid explanation, truly.

Now, we will, notwithstanding, confess, that for these people, or those who dream of lucky numbers in the lottery (for spirits do sometimes dabble in the stocks and the wheel)—for these to be converts is very natural; but how a woman in her senses can refer this to clairvoyance, we own extremely puzzles us, who take the myriads of unfulfilled prophecies as sets off against credulity.

"If dreams are essentially prophetic," says Evelyn, "why are they not all fulfilled? if one is not fulfilled, how know we if *all* will not be equally fallacious. The argument for the prophetic nature is the shallow post hoc ergo propter hoc of the sophist. On the occurrence of any important event, all the auguries and dreams which bear the slightest resemblance to a prophecy are stretched and warped to suit the superstition. Yet the fulfilment *may be* a consequence—

"1. Through the effect of imparted impetus.
"2. Foresight—study of events and character.
"3. Constant thinking on a subject.
"4. Mental impression."

Asserting, therefore, our full and sincere confidence in the goodness, wisdom, and omnipresence of the Deity, we cannot yet coincide with the sensible and serious writer on dreams, "that we must see that there has been a special design of Providence;" and that "they have conduced to infuse, in the absence of revealed truth, the sense of a spiritual and *prescient* power and of a future life."

To account for our heroism when we dream of a spirit, Mrs. Crowe coolly assures us that "spirit is not afraid of spirit." Dog will not eat dog.

The cases of "Double Dreaming" are cited as a sort of magnetic sympathy. Thus, Dr. Binns is quoted, where he writes of a gentleman who dreamed he pushed violently against a door: the same moment the people in that room were extremely alarmed by a violent pushing. This is very absurd, especially as "Nothing came of it."

Regarding trance and transmigration, the "intellectual waking with organic sleep," of Rozencrantz, it were futile to attempt to controvert the credulous proselyte who believes because people have *affirmed*, like Santa Teresa and Emanuel Swedenborg, that they have been in another world and seen departed spirits. The condition of Catalepsy, however, is one of deep pathological interest, as, indeed, are all the prototypes of sleep. We will, therefore, quote fragments from the chapter on these subjects in the "Philosophy of Mystery."

"The most impressive conditions of the mind are these:—

| | |
|---|---|
| Unconscious and passive | *Sound sleep.* |
| Conscious, yet passive | *Dreaming.* |
| Conscious and willing, yet powerless | *Night-mare.* |
| Unconscious, yet active | *Somnambulism.* |
| Unconscious, motionless, sensationless | *Deep catalepsy.* |

"In some cases of trance, the rosy colour of the lips and cheeks will not fade; in others, they are pale and bloodless, the body becomes cold as marble, the pulse often imperceptible, and the vapour of breathing on a polished surface alone distinguishes the still living being from the perfect work of the sculptor. I have, however, had patients who were rosy when they fell asleep, but became pale about the end of the second day. Girls often smile sweetly in deep catalepsy, but the countenance will become anxious as waking approaches; and this must ever excite suspicion.

"Previous to the cataleptic acme, girls are often maniacally violent, and will then suddenly regain their temper and their reason. They will sit and play with their fingers, in a sullen mood, and the power of motion, speech, and other acts of volition, may be alternately impaired or lost. In some, the sleep has been preceded by fits of lethargy, by lassitude, and inaptitude to exertion, and perhaps a propensity to sleep-walking. The decided state of catalepsy has begun in an epileptic convulsion. In all, I think, I have seen combined with this disorder, irregular determination of blood; in one case, where the taste and smell were gone for four or five months, the climax was suicide.

"The countenance is almost always placid in cataleptic sleep; the eyes being turned up, the pupils dilated, but the eyelids closed. If the fit be the result of sudden fright, the features will remain as they were at the moment, the eyelid fixed, but the pupil usually sensible. The joints and muscles are pliable, and may be moulded to any form; but they remain in that position as rigidly fixed as the limbs of a lay-figure, insensible to all stimuli—beating, tickling, and pricking.

"I have seen patients lapse into a state of catalepsy *in a moment*, without a struggle. I saw, during one of my visits to the asylum in Hoxton, a maniac, who often, in the midst of his occupation, became instantaneously *a statue;* leaning a little forward, one arm lifted up, and the index-finger pointed as at some interesting object; the eye staring and ghastly, and the whole expression as of one rapt in an ecstasy of thought or vision.

"The waking from a trance, like the recovery from the asphyxia of drowning, is painful. It is attended by a sensation as of the pricking of needles, and a struggle, and the hand is almost invariably placed firmly *over the heart*, as if its actions were a painful effort to overcome congestion. In some cases, a purple hue will suddenly suffuse the cateleptic body; the limbs are then extremely rigid, but become pliant when the healthy tint is restored.

"Hunger is unusually intense when the patient awakes. The usual duration of the fit is from twenty to forty hours. The return of volition is commonly marked by perspiration; this premonitory sign is often followed by a piercing shriek, as in the case of night-mare, and indeed, in a slight degree, of an infant's cry as soon as it is born."

Of Wraiths and Doppelgangers—people appearing out of their body—Mrs. Crowe says she has an "embarras des richesses,"—she is dying of too much sweet. But what can we think of one who believes the story related by Dr. Werner, of a young nobleman who, in Paris, struck with his whip at his father's shadow, who was dying far away, and who, at the moment of death, exclaimed, "My God, he is striking at me with his whip!"

In addition to the two hypotheses of ghosts, subjective and objective, Kieser it seems has a third, the magnetic influence of the seen over the seer—the reverse of the Hohenlohe influence—a projection of an astral spirit from its ponderable body. While they take all this trouble to explain, they again unconsciously refer to diseased perceptions, that the patient feels as if lying in two places at once; so that disease increases the locomotive power of this astral element. A sort of railroad or aërial machine is thus made out of the morbid man lying between the sheets.

And then Mrs. Crowe, as a sort of diversion, catches Dr. Ennemoser out in an inconsistency, in observing that self-seeing is an illusion; yet seeing the image of another is an objective reality. Why, if one astral element or magnetic influence can escape, why not another? She has him on the hip—set a thief to catch a thief. But every spirit, it is affirmed, is not *prepared* for this communion—however, this is a very pretty quarrel as it is, so we say no more.

It is amusing to observe the confidence and coolness of this learned lady in her probabilities. In explaining one of her ghost stories, she observes, "*Probably* the daughter was dreaming of her mother: projecting her astral element not only into her mother's room, but into her mother's soul." This beats the electric telegraph all to nothing. In another case, an old man, wishing to get home from a storm, projects his astral element to his arm-chair, and his daughter, anxious about him, projects hers, and so their *spirits* meet. But the old man gets soaked, and the daughter is in a fidget—*cui bono?*

Of a truth this sort of double-ganging is a capital invention: the lover at a distance from his mistress might project his astral element, or the maiden project hers. We must remember this element is *merely astral;* therefore, there can be no stain on the maiden's fair fame, even though, like Amina, she be found asleep and undressed on the couch of her lover. By Cupid, this is the sort of Platonism, for, being psychical, it is innocent. By this whimsical projection of soul Mrs. Crowe explains all optical illusions—apparitions, of course. Thus, when a little girl, who had been *smothered* for hydrophobia (!) was seen by her playmates, she had no doubt that "memory of past sport attracted the young soul to the scene of its former gambols." The vivid memory of the *living* girls could not, of course, raise a ghost.

We have next a fine story of some professors in the Carolina Colleges at Brunswick. One who died appeared to two others:—one of these has a swelling in his arm; "*because*," says Mrs. Crowe, "his physical nature was not adapted for spiritual intercourse." In this case the ghost paid many visits in its attempt to explain its wishes. A spirit should find a readier mode: but in good truth there is throughout so much straining to explain causes and reasons, that we close the page in pity.

And yet on the next our smiles are again excited, by the rationale of *speaking* ghosts, who, "by the magical will of the spirit, can simulate sounds." Fore George! we are prepared for wonders, more and more, by this new application of the science of projectiles. The force of Queen Anne's pocket-piece, or Mons Meg, or Warner's machine loaded with gun-cotton, were nothing to this projection of spirits a thousand miles in a second.

If Mrs. Crowe has looked into Dr. Thomson's Salverte, and thereupon where he writes of speaking heads, would she not have blushed thus to strain at a gnat, when she might, without trouble, have swallowed a camel.

The natural phonic mysteries of Memnon might suffice, but more modern art has even far eclipsed the ancients in its toys. Albertus

Magnus, it is true, in the thirteenth century, according to the learned editor of Salverte, "constructed an automaton, which both walked and spoke, answered questions and solved questions submitted to it." But we refer our readers to Dr. Thomson's work, if they wish to revel in the hearing of these unfoldings of optical and phonic mysteries.

We were proceeding in our glances—reading we can scarce call it—when we fairly burst into laughter at Mrs. Crowe's belief and assertion, that Bentham's objections can be at once removed by a projecting soul *conceiving* itself clothed in its body's breeches, and so represent itself to the eye or the constructive imagination of a seer. Now, we once heard of an Irishman, who, when the tailors struck for an advance of wages, suggested the avoiding of expense for clothes by getting them *ready made.* But here is an actual spirit that can *conceive* a pair of breeches. Why, a firm of such choice spirits would soon make Stultz shut up his shop. Now, in sober sadness, is it not deplorable that a woman's pen should thus endow the soul not only with the power of projecting itself, but even a pair of corduroys or kerseymeres? Truly, this is an *inexpressible* faculty.

"The future that awaits us."

Such is the heading of the last Night Side chapter of vol. i. Shall we discuss it?—for the subject is too solemn for ridicule. But we confess we have no faith in Mrs. Crowe's discretion. What can *she* write about it? Let us try to discover. We have read several pages, and cannot get into her subject yet. Stay, we see two words beginning with capitals —Tartarus and Elysium. Surely this must be interesting. So, we have a sort of statistical report. It seems that Tartarus and Elysium are very thinly populated, because the *very* wicked and the *very* good abound not on earth. It is into Hades that the *petty* sinners flock in crowds—and yet it is said, heaven and hell are not places, but states; but a body must have locality and space. Mrs. Crowe thinks this Hades is not far off from earth—(is not earth itself, just now, Hades?)—to this the soul is still attracted by the magnet, and where it plays about, revisiting the earth when it will; which, by the way, Isaac Taylor terms a trespass. Now, considering the rarity of ghostly trespassers from Hades, we must say these ghosts are very contented beings, or that Hades is a very lenient state of purgatory. But let us use the rhabdomantic art, and, by our divining rod, find a grain of Mrs. Crowe's gold. There is some ingenuity in the disquisition on the nature of future punishment.

She believes celestial happiness consists in our being able to look on, and fully appreciate, and enjoy God's beautiful creation, as pure and simple beings. Hell consists in the negative of this—it is a state of desire ungratified to all eternity. The good will love each other's beauty and virtue, the bad loathe each other's depravity; and thus, the most intense feelings of happiness and misery be produced. But it seems we may improve even here, undergoing a sort of purifying process—the purgatory of the Romanist.

Now, although gained from the visionary and the somnambulist, this is not an inconsistent conjecture. But then, Mrs. Crowe sets herself to argue that the revelations (*we* should call them ravings) of frenzy, are to be taken as celestial visitations—glimpses of futurity: and for these visitations, women, she says, are the most fit, because woman is the most *receptive*—man the most *productive*. So that women even on earth are indeed angelic.

So woman is the great fount of revelation; whether Joanna Southcote or St. Teresa be the coryphæa, we are not told. It is she who has seen heaven : the apostles of the modern church can only read and form conjectural conclusions, in which, perchance, like the last chapter in Rasselas, nothing is concluded. Then of what avail is collegiate learning?—of what use the courses of divinity from the lips of sage and solemn professors, if a poor cataleptic is to unfold and map out the topography of heaven, and a wildish woman show us the way thither. We, chaperoned by our sable sister, have thus traced the spirit to its Hades, from whence she now believes millions wish to revisit earth, but they cannot. Why? —because they must first find out a body endowed with a congenial spirit, a magnetic rapport or polarity—"the opening of the eye" of the prophet—and then, presto ! the flight is easy.

On the rhapsody that follows the really interesting cure of Miss Lee, in explanation of magnetic rapport, it would be useless to dilate; for when we see the halo of light, which silly girls affirm surrounds the heads of their magnetizers, "strong magnetic men," according to the Corvine zoology, identified with that around the brows of saints, having been well acquainted with the sanctity of some of these rude impostors whose secret doings would *shame* the saintship of an anchorite, we confess we blush at the profanation of such an unholy comparison.

Mrs. Crowe is welcome to scribble on about her æther, which connects the members of creation one with another, but let her not presume to liken the blue light only existing in the fancy of a wanton girl with the glory which sacred record has placed around the holy brows of saints and martyrs.

We are at the end of the first volume, and we rejoice at it, for we wished to sift it completely. To analyze, however, the *supersensuous* domain of nature, the *all-pervading æther*, the questions of moral *weight* and moral *darkness*, the paradox that spirits most in love with earth and its creatures most frequently visit it—many of these loving ghosts having been cut off by assassination,—all this, we confess, far surpasses our limited capacity. Even the shade of Salverte, with the aid even of his learned translator would not be Œdipus enough to solve the riddles of our sable sphinx.

The second volume opens with the bold challenge that the power of the will can do anything. It gives us the power of hearing a sound not audible, of course of seeing things invisible : it is "the sense conveyed without words !" We hope our readers understand this—we do not.

After implicit confidence in the power of the magi to assume any form they will, of wraiths in wet clothes appearing many miles from the scene of drowning, we come to the discussion as to *how long* after separation of soul and body a wraith or death-fetch can appear. And here we have a flaw in the indictment which puzzles even Mrs. Crowe, regarding the interaction of souls. Although she boldly avers that within a day or two the just departed soul may flit, yet she is compelled to confess that when two or three days have elapsed ere the wraith appears, it must be *subjective*—that is, spectral illusion; this is followed up, however, by an "except" and an "unless," indicating the wavering state of the lady's mind on this point.

She soon, however, gets over this puzzle, with the aid of Dr. Passavent, by whose *far-working* theory and the nerve spirit of her somnambulists, she reconciles the reality of all the wraiths and fetches that have ever been blazoned in the pages of natural magic.

Then we have a touch of the evil eye, and a sort of diamond-cut-diamond story, of Rousseau's attempting to kill a toad with his eye, when the thing got the whip-hand of him, and by the power of its will, through its eye, threw Jean Jaques into a fainting fit!

Those confiding ghosts which "seek prayers from the living" put on various forms—sometimes a shadow, sometimes a light, as the case may be. They lay wait for some poor creature with whom they can establish or support a "magnetic relation," and then they play away at us. No one is safe; we are all liable to this *vis*, at any time and in any place. There is a most extraordinary story of a female prisoner, Eslinger, in Weinsburg, attested by doctors, magistrates, &c. &c., who was for a long period nightly visited by a ghost, which ladies who slept in the cell saw, heard, and felt. Here must have been some gross imposition, or blind credulity—probably both—the woman having confessed that the shade had caused her to commit the crime for which she was incarcerated.

Regarding troubled spirits, Mrs. Crowe has discovered—how we cannot conjecture—that unhappy souls have cold hands, happy souls warm ones. And then she has *heard of* three cases of continual intercourse between deceased couples; but as to what they say or do we are left in the dark. The third case of the lady is decidedly one of bigamy—although remarried, she constantly received visits from her first husband. However, *we* will not interfere.

In her chapter on haunted houses, Mrs. Crowe quotes Pliny's letter regarding that of Athenodorus, but blinks, very unfairly, those of Tedworth, Cock-lane, Woodstock, &c. &c., the mysteries of which were at length fairly exposed.

Of a very wondrous Poltergeist or racketing ghost, at the castle of Prince Hohenlohe, in Silesia, Mrs. Crowe concludes that the only key to the mystery was the finding of a split skull lying by a sword and without a coffin.

Of possession, also, very extraordinary and even respectably-attested stories are told, calculated to alarm the timid with the dread of being possessed by these worrying devils. Indeed, if these tales were believed, and the truth of demoniac possession and witchcraft established, we shall have Chief Justice Denman sit, like Sir Matthew Hale, to condemn the old Hecate, and Bishop Blomfield preach before Queen Victoria, as Jewell did before Elizabeth, telling her that great misery was caused by monstrous witcheries. But "enough," we fear our readers have already exclaimed. Let us, therefore, give a parting glance at the volumes whose titles we have annexed.

Mrs. Crowe's book is certainly well written; its legends and its rhapsodies well wrapped up and amusing; but it is less interesting than the "Mysteries of Udolpho," or the "Romance of the Forest." Mrs. Radcliffe is candid, she *calls* her books romances, and developes very strange mysteries. Mrs. Crowe of course calls *her* books philosophy, and yet she *elucidates* nothing—the Night Side of Nature would still have been in darkness had we not perused books of true scientific and physiological research, on two of which we have freely commented, as they reflect from afar a light on her obscurity.

The work of Mons. Salverte indicates immense research, displaying to the reader an association of incidents and elucidations of natural and artificial mysteries, as complete as the gigantic march of science can enable us to compass. It will be seen how comprehensive is the disquisition when we enumerate its subjects—credulity, marvellous histories, magic, thaumaturgy, oracles, hieroglyphics, acoustics, optics, hydrostatics, secret working of miracles, narcotics, perfumes, gases and odours, ointments, occult medicine, poisons, philters, meteorology, phosphorescence, —hence must the perusal be deeply interesting, especially to the student of natural and experimental philosophy. It is true, Salverte was too much in love with matter of fact, and has, therefore, warped somewhat the acknowledged miracles of Holy Writ to suit his purpose; but his translator has been too busy in their almost complete suppression, and has indeed thus emasculated his volumes.

Dr. Thomson's translation is made with fidelity and freedom: but there is a very serious question attached to the appearance of his two large volumes: Was the translation called for? Curiosity, it is true, is gratified even to satiety, but still the work is not productive of that full conviction which a scientific treatise, professing to elucidate psychology by unhooding imposture, should be. The realm of "natural magic" had been amply surveyed by Sir David Brewster, whose concise, beautiful, and logical little book formed so agreeable a contrast to the hotch-potch "Demonology" of Sir Walter Scott.

"The World of Wonders" is a sad misnomer. The book is a mere unintellectual catalogue of curiosities, jumbled together in very beautiful confusion; evidently to make a book. Many of the stories have been often told, and the natural phenomena far more scientifically recorded. And as to "Wonders," we should wish to know if such subjects as "*negroes—verbal errors—male and female—education of children,*" &c., can possibly be brought under this category. Then, as to the points of interest in the work, the only one is the editor's name; and when we have amended the title page thus, "for POYNTZ read GORE," we shall dismiss to obscurity the "World of Wonders."

The plan of the "Communications with the Unseen World" appears to be, although the author professes otherwise, a slight, and, of course, unconscious imitation of the "*Philosophy of Mystery,*" of *Mr. Dendy.*

Mr. Sheppard's book on "Dreams" is a very candid exposé and analysis: its chief aim being to establish the moral and religious *utility* of dreaming, "affording," as the author professes, "auxiliary arguments for the existence of spirit—for a separate state, and for a particular providence."

Admiring the author's motives, we differ from him in very many points. Mr. Sheppard is no physiologist, and therefore he cannot *elucidate* the mysteries of psychology. His sensible mind and mature reflection have, however, produced a small volume of very clear and intelligible writings; yet his arguments are only, as they must be, abstract propositions; and his definitions somewhat vague. Thus he observes, "If not miracles, they are at least secret actings of Providence." There is this secret acting, this divinity *ever* stirring within us; but that only is a *miracle* which is a rare deviation from a gigantic law.

The "Philosophy of Mystery," from which we have so largely quoted, is from the pen of a physiologist and a psychologist of distinguished reputation. The work is written to reconcile, if possible, the discrepancy of two classes of philosophers, *pathologists* and *divines*, illustrating by facts and deductions those beautiful and eccentric phenomena which are resulting from physical causes, and separating from the sacred record, which we can conceive only through our belief and faith in the revelations of Holy Writ; vindicating the sacredness of Scripture miracles, and weeding philosophy of those false data which tend to hoodwink the mind, and prevent the elucidation of truth. The work in question is of the highest order in an intellectual point of view, and places the author in an advanced position among the medical philosophers and psychologists of the present day—that class of thinkers who (perhaps improperly) conceive that the science of medicine has far higher and nobler functions than that of teaching the practitioner the art of wielding with effect the agents of the *Materia Medica*.

